# Tumoricidal Potential of Novel Amino-1,10-phenanthroline Derived Imine Ligands: Chemical Preparation, Structure, and Biological Investigations

**DOI:** 10.3390/molecules25122865

**Published:** 2020-06-22

**Authors:** Kollur Shiva Prasad, Renjith Raveendran Pillai, Chandan Shivamallu, Shashanka K. Prasad, Anisha S. Jain, Sushma Pradeep, Stevan Armaković, Sanja J. Armaković, Chandrashekar Srinivasa, Sharadadevi Kallimani, Raghavendra G. Amachawadi, Veena Malligere Ankegowda, Najat Marraiki, Abdallah M. Elgorban, Asad Syed

**Affiliations:** 1 Department of Sciences, Amrita School of Arts and Sciences, Amrita Vishwa Vidyapeetham, Mysuru 5700 26, Karnataka, India; 2 Central Polytechnic College, Vattiyoorkavu, Trivandrum 695013, Kerala, India; renjithkadavoor@gmail.com; 3 Department of Physics, T.K.M. College of Arts and Science, Karicode, Kollam 691 005, Kerala, India; anishasjain@jssuni.edu.in; 4 Division of Biotechnology and Bioinformatics, School of Life Sciences, JSS Academy of Higher Education & Research, Mysuru 570 015, Karnataka, India; shashankaprasad@jssuni.edu.in (S.K.P.); sushmap@jssuni.edu.in (S.P.); 5 Department of Physics, Faculty of Sciences, University of Novi Sad, Trg D. Obradovića 4, Novi Sad 21000, Serbia; stevan.armakovic@df.uns.ac.rs; 6 Department of Chemistry, Biochemistry and Environmental Protection, Faculty of Sciences, University of Novi Sad, Trg D. Obradovića 3, Novi Sad 21000, Serbia; sanja.armakovic@dh.uns.ac.rs; 7 Department of Biotechnology, Davangere University, Shivagangotri, Davangere 577 007, Karnataka, India; chandru.s@davangereuniversity.ac.in; 8 Department of PG Studies & Research in Food Technology, Davangere University, Shivagangotri, Davangere 577 007, Karnataka, India; sharada_k@davangereuniversity.ac.in; 9 Department of Clinical Sciences, College of Veterinary Medicine, Kansas State University, Manhattan, KS 66506, USA; agraghav@vet.k-state.edu; 10 Department of Chemistry, Bangalore Institute of Technology, K.R. Road, V V Puram, Bangalore 560 004, Karnataka, India; veenamdy12@gmail.com; 11 Department of Botany and Microbiology, College of Science, King Saud University, P.O. Box 2455, Riyadh 11451, Saudi Arabia; najat@ksu.edu.sa (N.M.); aelgorban@ksu.edu.sa (A.M.E.); 12 Centre of Excellence in Biotechnology Research, King Saud University, P.O. Box 2455, Riyadh 11451, Saudi Arabia

**Keywords:** Phenanthrolin-5-yl, selective cytotoxicity, MD simulations, computational analysis

## Abstract

Herein we report the synthesis and structural elucidation of two novel imine-based ligands, 2-(1,10-phenanthrolin-5-yl)imino)methyl)-5-bromophenol (PIB) and *N*-(1,10-phenanthrolin-5-yl)-1-(thiophen-3-yl)methanimine (PTM) ligands. An in vitro cytotoxicity assay of the synthesized molecules was carried out against breast, cervical, colorectal, and prostate cancer cell lines as well as immortalized human keratinocytes. The observations indicated that both the molecules possesses dose-dependent selective cytotoxicity of cancer cells with no detrimental effect on the normal cell lines. Furthermore, the detailed computational analysis of newly synthetized ligands (PIB and PTM) has been conducted in order to identify their most important parts from the perspective of local reactivity. The IC_50_ values of PIB treatment on MCF-7, HeLa, HCT-116 and PC-3 were 15.10, 16.25, 17.88, 17.55 and 23.86 micromoles, respectively. Meanwhile, the IC_50_ values of PTM on MCF-7, HeLa, HCT-116, PC-3 and HaCat were observed to be 14.82, 15.03, 17.88, 17.28 and 21.22 micromoles, respectively. For computational analysis, we have employed the combination of Density Functional Theory (DFT) calculations and MD simulations. DFT calculations provided us with information about structure and reactivity descriptors based on the electron distribution. Surfaces of molecular electrostatic potential (MEP) and averaged local ionization energy (ALIE) indicated the sites within studied molecules that are most reactive. These results indicated the importance of nitrogen atoms and OH group. Additionally, the values of bond dissociation for hydrogen abstraction showed that both molecules, especially the PTM, are stable toward the influence of autoxidation mechanism. On the other side, MD simulations gave us an insight how ligands interact with water molecules. Namely, the radial distribution functions (RDF) indicated that the hydrogen atom of the OH group in the case of the PIB has the most pronounced interactions with water.

## 1. Introduction

Nitrogen-containing ligands are constantly gaining attention of chemists due to their stable chelating property [1]. Furthermore, these types of molecular entities find wide array of applications. [2,3]. For instance, they provide opportunities for tuning the electronic properties of metallic centers, thereby increasing the catalytic properties of the substrate chirality [4]. Because of a rigid structure supported by the central ring, 1,10-phenanthroline and its derivatives received enormous interest in the synthesis of metal complexes and which occurs faster due to contiguity of nitrogen atoms in the aromatic rings. Concerning the application, 1,10-Phenanthroline and its derivatives in biology, they demonstrated potential antimicrobial and anticancer activities [5,6].

It is noteworthy that phenanthroline derivatives are of exceptional scientific interest owing to their biomedical significance, with established tumoricidal and antimicrobial activities [6]. Imines, such as Ketamine, azomethine and aldamine, have also demonstrated significant anti-proliferative properties so that they have been considered potent clinical candidates for the treatment of cancer [7]. Similarly, 1,10-phenanthroline derived molecule, *m*-bispyridylphenyl cyclometalated-1,10-phenanthroline, showed anti-cancer activity higher than the cisplatin positive control [8]. In a recent report, it was concluded that the Palladium(II) and Platinum(II) containing secondary ligands complexes derived from 9,10-phenanthroline showed promising anti-breast and anti-ovarian cancer activity with IC_50_ values <60 μM as compared to IC_50_ values >30 μM for cisplatin, for both the cell lines [9]. Furthermore, a metal-mediated coordination compound containing 1,10-phenanthroline ligand complexes were reported to show potent DNA binding and anti-cancer activity in human laryngeal carcinoma (Hep-2) cells [10]. In what may be considered the sole study reporting mechanistic evaluation of the anti-tumorigenic potential of phenanthroline derived ligand complexes conducted on hepatoma cell lines (BEL-7402, Huh-7 and Hep G2), Zhang, Y. et al. (2013), concluded that the cytotoxicity was mainly due to apoptosis induced after a cell cycle arrest at the G_0_/G_1_ phase. Additionally, depolarization of the mitochondrial membrane was observed due to a decrease in the mitochondrial membrane potential [11]. Although the findings reported are satisfactory, the numbers of reported activities are inadequate. It may be deemed imperative to understand the cytotoxic behavior of the phenanthroline derivatives across different types of cancer as well as normal cell lines along with validation of the mechanistic route in an attempt to identify potent phenanthroline derived drug candidates to qualify for in vivo and pre-clinical evaluation.

## 2. Results and Discussion

**Scheme 1:**
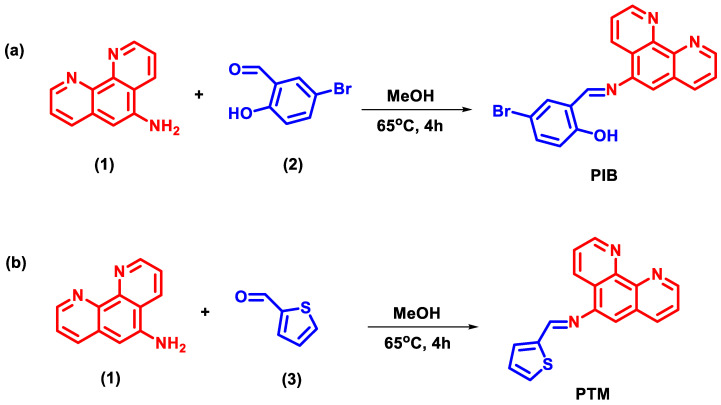
Synthetic route of obtaining (**a**) PIB and (**b**) PTM ligands.

The condensation reaction of 1,10-phenanthrolin-5-amine with 5-bromo-2-hydroxybenzaldehyde and thiophene-3-carbaldehyde yielded PIB and PTM ligands, respectively, as depicted in the Scheme 1. The structural details were elucidated by ^1^H, ^13^C NMR and mass spectral analysis. In the proton NMR spectra of PIB and PTM (Figures S1 and S4), the multiplet signals corresponding to the aromatic protons appeared in the region 8.03–6.58 ppm in PIB and 7.84–6.08 ppm in PTM, respectively. A singlet observed at 8.94 ppm in PIB and at 8.99 ppm in PTM corresponds to imino proton (CH=N). The corresponding ^13^C NMR spectra are provided in Figure S2 and S5, which showed imine carbon atom signal at 167.75 and 167.51 ppm in PIB and PTM ligands, respectively. In addition, the aromatic carbon atoms were observed between 126 and 139 ppm. Their formation was further confirmed by mass spectral analysis, where the respective molecular ion peaks for PIB and PTM were found at *m*/*z* = 377.1 and 289.8 (Figures S3 and S6).

### 2.1. Dose-Dependent Selective Cytotoxicity

The novel imine-based ligands, PIB and PTM were treated for 24 h on breast, cervical, colorectal, and prostate cancer cell lines to check for their tumoricidal activity. The treatment was carried using varied concentration (2–60 μM) of chemicals on all the cell lines. Additionally, toxicity of the synthesized molecules was also tested upon using the normal human fibroblast cell line, HaCat. Interestingly, both the ligands, showed selective dose-dependent anticancer activity across all the tested cancer cell lines.

**Figure 1:**
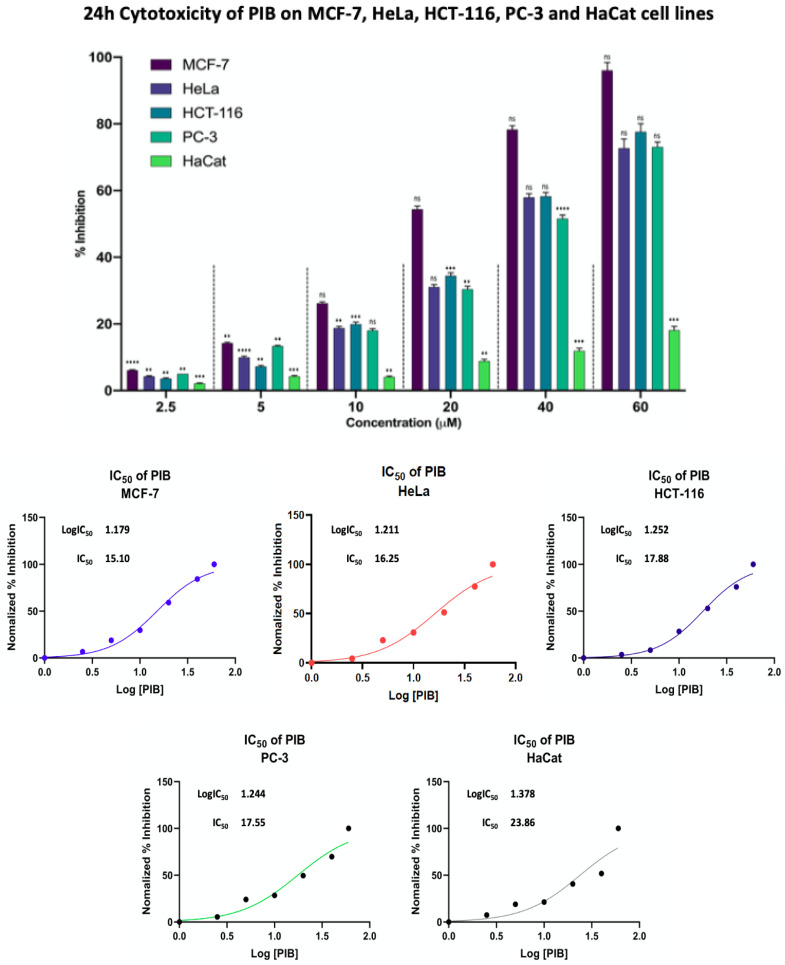
PIB showed highest cytotoxicity in breast and colorectal cancer cell lines. Upon treatment of PIB for 24 h on breast (MCF-7), cervical (HeLa), colorectal (HCT-116) and prostate (PC-3) cell lines, % inhibition of ~97% of MCF-7 and ~80% of HCT-116 cells was observed, while ~75% of HeLa and PC-3 was recorded. The anti-cancer activity observed in all the cell lines as dose-dependent. PIB showed ~20% growth inhibition in normal human keratinocyte, HaCat, cell line. In addition, the number of cells were much lesser in the PIB treated groups. *n* = 3, mean ± SEM, *p value* of <0.05 was considered to be significant, ** p ≤ 0.01, *** p ≤ 0.0001, **** *p* ≤ 0.00001 and *ns* = not significant. The IC_50_ values of PIB on MCF-7, HeLa, HCT-116, PC-3 and HaCat were observed to be 15.10, 16.25, 17.88, 17.55 and 23.86 micromoles, respectively.

**Figure 2:**
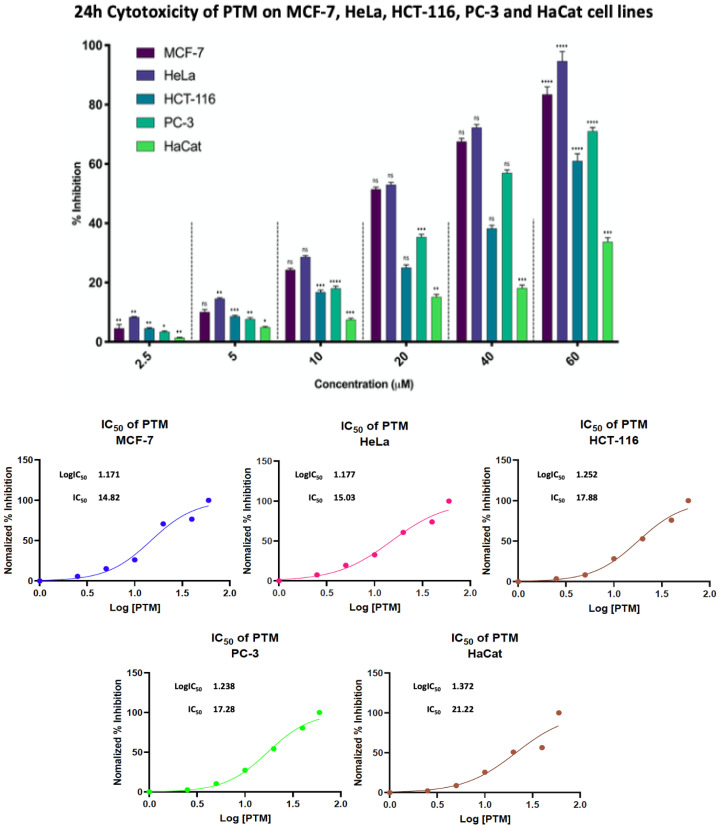
PTM showed highest cytotoxicity in cervical and breast cancer cell lines. Upon treatment of PTM for 24 h on breast (MCF-7), cervical (HeLa), colorectal (HCT-116) and prostate (PC-3) cell lines, % inhibition of ~96% of HeLa and ~82% of MCF-7 cells was observed. Additionally, PTM treated HCT-116 and PC-3 showed % inhibition of ~60% and ~70%, respectively. The anti-cancer activity observed in all the cell lines was dose-dependent. PTM showed ~40% growth inhibition in normal human keratinocyte, HaCat, cell line. In addition, the number of cells were much lesser in the PTM treated groups. *n* = 3, mean ± SEM, *p value* of <0.05 was considered to be significant, * p ≤ 0.05, ** p ≤ 0.01, *** p ≤ 0.0001, **** *p* ≤ 0.00001 and *ns* = not significant. The IC_50_ values of PTM on MCF-7, HeLa, HCT-116, PC-3 and HaCat were observed to be 14.82, 15.03, 17.88, 17.28 and 21.22 micromoles, respectively.

The 24 h treatment of PIB showed great inhibition of breast and colorectal carcinoma cell lines, MCF-7 and HCT-116, respectively. While the ligand treatment at its highest concentration inhibited 96.05% of MCF-7 cells, an inhibition of 77.61% was observed in the HCT-116 cells. Furthermore, the HeLa and PC-3 cell lines showed 72.73% and 73.06% inhibitions, respectively, at the highest dose of PIB (Figure 1). PIB treatment of MCF-7 and HCT-116 showed IC_50_ values of 15.10 μM and 17.88 μM, respectively. The observed IC_50_ of PIB treatment on HeLa was 16.25 μM while the same for PC-3 was 17.55 μM. Meanwhile, highest concentration (60 μM) treatment of the PTM ligand showed anti-proliferative activity up to the tune of 83.45%, 94.65%, 61.02% and 71.04% in the respective MCF-7, HeLa, HCT-116 and PC-3 cell lines (Figure 2). This is mainly because of the potent action of the PTM molecule against breast and cervical cancer. PTM treatment of MCF-7 and HCT-116 showed IC_50_ values of 14.82 μM and 17.88 μM, respectively. The observed IC_50_ of PTM treatment on HeLa was 15.03 μM while the same for PC-3 was 17.28 μM. Surprisingly, both the novel ligand molecules synthesized were not promisingly toxic to the normal human fibroblast cells, with the highest doses of PIB and PTM inhibiting only 18.12% and 33.74% of the HaCat cells, respectively, at 60 μM concentration and showed a higher IC_50_ of 23.86 μM and 21.22, as compared to the treatment on cancer cell lines.

In a recent investigation conducted by Zasheva et al. (2019), it was concluded that the molecular complexes comprising of phenanthroline moiety were potent anti-breast and anti-prostate cancer candidates [12]. Similar observation was made by Kaloyanov et al. (2011) where the novel molecular complexes containing 5-amino-1,10-phenanthroline exhibiting cytotoxicic potential against the human laryngeal carcinoma (Hep2), human liver cancer (HepG2) and Human glioblastoma multiforme brain tumor (8-MG-BA) cell lines without inhibiting the in vitro human embryonic stem Lep3 cell growth [13]. All of which established the selective cytotoxicity of the novel amino-1,10-phenanthroline derived imine ligands, PIB and PTM, as observed in the current study. However, the above observed IC_50_ values of PIB as well as PTM when compared against that of the standard drug for cancer, Cisplatin, are high. Cisplatin has a reported IC_50_ values of 3.2 μM in MCF-7 [13], 10.07 μM in HeLa [14], 4.2 μM in HCT-116 [15], 3.3 μM in PC-3 [16] cell lines. However, the associations of cisplatin treatment with nausea, nephro-, cardio-, hepato- and neuro-toxicities are well known [17]. Therefore suggesting the PIB and PTM molecules maybe considered to be safer alternatives to cisplatin and thus endorsing the need for detailed investigation into their mechanistic behaviors.

### 2.2. Local Reactivity Properties of Investigated Structures

MEP and ALIE surfaces are widely used descriptors for prediction/confirmation of reactive properties. These surfaces are obtained by mapping of their values to the electron density surface due to their relation with electron density, these two descriptors are considered to be fundamental quantum-molecular descriptors. Regarding the MEP, neglecting the effects related to polarization and nuclear rearrangement as a consequence of presence of a unit test charge at distance *r*, the MEP, *V*(*r*), can be defined by the following Equation (1):

(1) $$V\left(\vec{r}\right)=\sum \frac{Z_{A}}{\left|R_{A}-\vec{r}\right|}-\int \frac{\rho \left(\vec{r}’\right)}{\left|\vec{r}’-\vec{r}\right|}\;d\vec{r}$$

*ρ*(*r*’) denotes molecule’s electron density, while summation is performed over all nuclei *A* with charge *Z*_A_ and coordinate *R*_A_. *V*(*r*) is potential exerted at coordinate *r* by nuclei and electrons. The sign of *V*(*r*) at any point indicates whether the effects of electron or nuclei is dominant [13,14], and for the practical applications it is useful to identify the molecule parts with the highest magnitudes of MEP. Precisely these locations are the most reactive, from the perspective of electrostatic interactions.

MEP is also frequently employed to identify the molecular sites prone to electrophilic attacks, although it is not the best descriptor for that purpose. To get more reliable prediction of molecular sites prone to electrophilic attacks, it is better to use the ALIE descriptor, since it provides the information about the energy required to remove an electron from a certain point around a studied molecule. ALIE is defined as a sum of orbital energies weighted by the orbital densities [15,16], which is described by the Equation (2):

(2) $$I\left(r\right)=\sum_{i}\frac{\rho_{i}\left(\vec{r}\right)\;\left|\varepsilon_{i}\right|}{\rho \left(\vec{r}\right)}$$

$\rho_{i}\left(\vec{r}\right)$ represents the electronic density of the *i*-th molecular orbital at the point $\vec{r}$, $\varepsilon_{i}$ represents the orbital energy, while $\rho \left(\vec{r}\right)$ is the total electronic density function.

**Figure 3:**
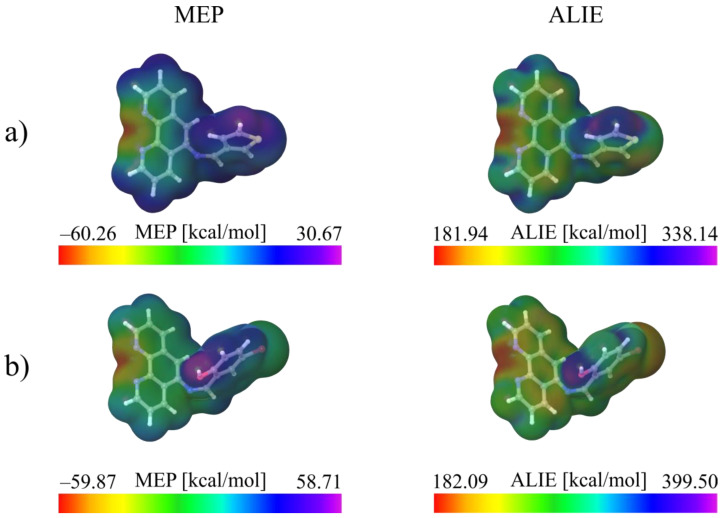
MEP and ALIE surfaces of (**a**) PTM and (**b**) PIB ligands.

Taking into account the strengths and weaknesses of MEP and ALIE descriptors, one easily concludes that the best solution when it comes to the determination of reactive properties of newly synthetized molecules is to apply both descriptors. This has been done for the investigation of ligands synthetized in our present study and the results are presented in Figure 3.

According to the results presented in Figure 3, the lowest values of MEP remains same for both the ligands, indicating that the both ligands are with practically the same affinity for interaction with positively charged parts of other molecules. It can be also stated that both ligands are also with very low MEP values, equal to ~−60 kcal/mol. On the other side, there is high difference in the highest MEP values; namely the highest MEP value of PIB ligand is almost two times higher than the highest MEP value of PTM ligand. Distribution of MEP values is practically the same for both ligands; the lowest MEP values are located in the near vicinity of N19 and N20 atoms, while the highest MEP values are located in the near vicinity of OH group of PIB.

On the other side, both ligands have practically the same lowest ALIE values, indicating that both ligands have practically the same sensitivity toward the electrophilic attacks. In terms of sensitivity toward the electrophilic attacks, the lower the ALIE value is, molecule is more sensitive toward the electrophilic attacks. Both ligands are characterized by the ALIE values of ~180 kcal/mol, indicating that both of them could be significantly sensitive toward the electrophilic attacks. The lowest ALIE values are located again in the near vicinity of nitrogen atoms N19 and N20. In terms of the highest ALIE values, there is a very significant difference; the PIB ligand is characterized by the much higher maximal ALIE value (higher for more than 60 kcal/mol), located in the near vicinity of OH group. This indicates that PTM ligand is more reactive, since lower amount of energy is required for the removal of electrons.

Analysis of MEP and ALIE surfaces undoubtedly confirmed the importance of nitrogen atoms of phenanthroline moiety and OH group, in terms of reactivity and potential to interact with other molecular structures. Kumar et al. [17] have reported the investigation of organic cocrystals involving the phenanthroline-5,6-dione derivative and aryloxyacetic acid. Their results indicate that the interactions between these two molecules are involving nitrogen atom of the phenanthroline derivative. Using the concept of Fukui functions, Cobos-Murcia et al. [18] have confirmed the crucial reactive importance of nitrogen atoms of phenanthroline moiety, while investigating electrochemical polymerization of 5-amino-1,10-phenanthroline to selected substrates. To investigate the adsorption of phenanthroline and its CH3-, S- and CN-substituted derivatives on the Au (111) surface, Vinodha et al. [19] also confirmed the importance of nitrogen atoms of phenanthroline by considering frontier molecular surfaces and electron density difference. These and other articles indicate that our findings regarding local reactivity of PTM and PIB are in agreement with the previously reported results.

### 2.3. Bond Dissociation Energies for Hydrogen Abstraction

**Figure 4:**
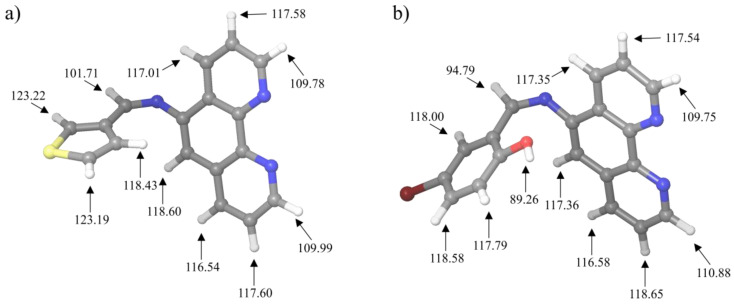
H-BDE values of (**a**) PTM and (**b**) PIB.

By using the concept of H-BDE, one is able to identify the sites of molecules that could be sensitive toward the autoxidation mechanism. Thanks to the established connection between the autoxidation and the certain interval of values of H-BDE parameter, using DFT calculations in this work we have located the sites of PTM and PIB ligands that could be sensitive toward this important mechanism. The H-BDE parameter has been calculated and presented in Figure 4.

Autoxidation is correlated with H-BDE [20,21], and it was established that if H-BDE parameter takes a value in a range between 70 and 85 kcal/mol, the studied molecule could be sensitive toward the autoxidation mechanism [22]. It should be also taken into account that H-BDE values in a range between 85 kcal/mol and 90 kcal/mol might also be of interest for sensitivity toward the autoxidation. Results presented in Figure 4 indicate that there are significant differences in the lowest H-BDE values. Namely all of the H-BDE values in case of the PTM ligand are much higher than the upper border value of 90 kcal/mol. On the other side, the lowest H-BDE value of PIB ligand is just below the upper threshold and is equal to 89.26 kcal/mol. The calculated value is referred to the O−H bond of the PIB ligand. This value indicates that PIB ligand is much more reactive in terms of autoxidation, which might have consequences when it comes to the possible pharmaceutical applications of molecules synthetized in this study.

It has been established that autoxidation mechanism presents one of the weakest links in the drug development process, because potential genotoxic impurities (PGI) could be formed precisely thanks to this mechanism [20,23]. Therefore, it is an imperative to be able to predict the drugs sensitivity toward the autoxidation mechanism. Both of the studied molecules in this work are with H-BDE values higher than 85 kcal/mol. Although the PIB has one H-BDE value just slightly below the upper border level of 90 kcal/mol, it is still much higher than the 85 kcal/mol, and such values are rarely found to be significant for the autoxidation mechanism [22]. Additionally, the corresponding hydrogen atom H35 of PIB has significant interactions with water, according to the results presented in the next chapter. The interactions between H35 and water molecules indicate that even if the autoxidation mechanism would be possible at this site, it would have to heavily compete with the influence of water. Therefore, all of the results indicate that pharmaceutical compounds based on the PIB and PTM should be stable in terms of “shelf life”.

### 2.4. Molecular Dynamics Simulations and Interactions with Water

**Figure 5:**
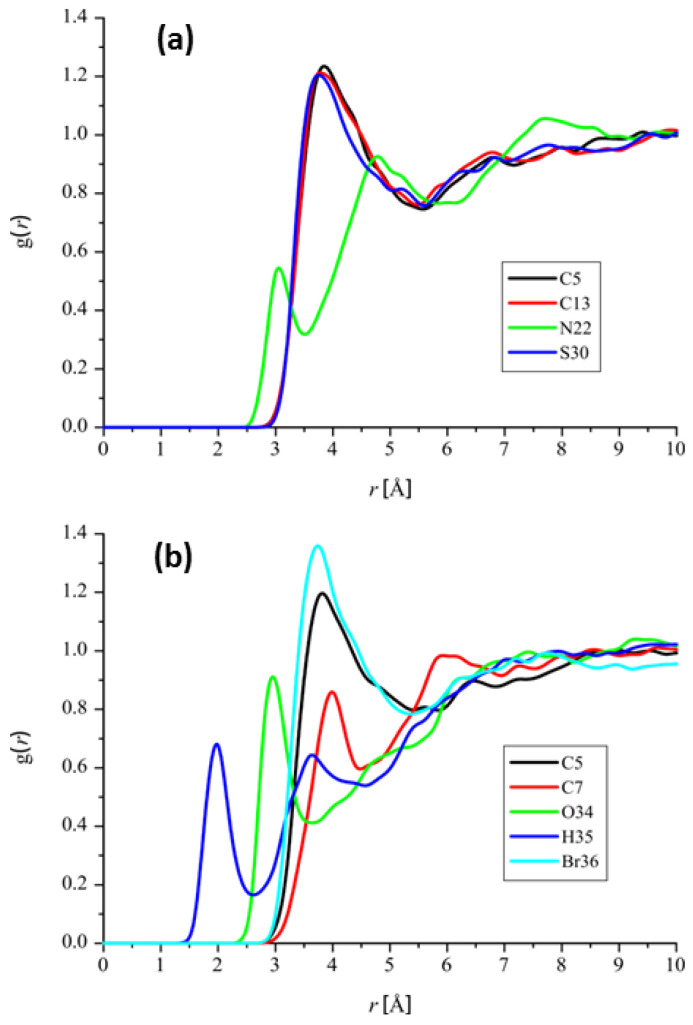
The most important RDFs of (**a**) PTM and (**b**) PIB.

MD simulations in this work were used in order to check which atoms of the PIB and PTM ligands have significant interactions with water molecules. The motivation for these simulations we find in a fact that aquatic medium is the most important one when it comes to the biochemical mechanisms. To identify the most important atoms from the perspective of interactions with water molecules, we used the concept of radial distribution functions (RDF), which provide the information about the probability density of finding a water molecule with respect to the distance between atoms of considered molecules (ligands PTM and PIB). The most important RDFs of PTM and PIB have been summarized in Figure 5.

Results presented in Figure 5 indicate that PIB ligand is more reactive in terms of reactivity with water molecules. Firstly, PIB possesses more atoms that have relatively significant interactions with water molecules. Secondly, maximal g(r) values are higher in cases of atoms of PIB and last but not least, maximal g(r) values are located at distances that are lower than in case of the PTM. With respect to the reactivity with water, it is especially important to mention the hydrogen atom of PIB’s OH group. Namely RDF of this atom is characterized with the curve whose maximal g(r) value is located at distance of around 2 Å, which corresponds to the strong interactions with water molecules. Another importance of the OH group in case of PIB ligand is shown by the fact that its oxygen atom is also characterized by the significant RDF, with high maximal g(r) value located at the distance lower than 3 Å. The highest maximal g(r) value is again reserved for one of the PIB’s atoms—in this case it is the bromine atom Br36.

The fact that PIB’s hydrogen atom H35 has significant interactions with water is particularly important. H-BDE value at the PIB’s H35 atom has a value which might indicate certain sensitivity toward the autoxidation mechanism. However, the sensitivity of the H35 toward the influence of water indicates that the autoxidation mechanism could not take part because of the competing influence of water. Competing influence of water versus autoxidation mechanism has been nicely demonstrated in case of the diazepam. According to the H-BDE values for diazepam (82.7 kcal/mol at the PBE level of theory), this particular molecule should have been sensitive toward the autoxidation; however experiments show the opposite, due to the competition with hydrolysis, as explained by Lienard et al. [24]. Taking into account the high H-BDE values and preference of PIB’s atom H35 toward the interactions with water, it is expected that these molecules could have suitable stability required for development of pharmaceutical products [25,26].

## 3. Materials and Methods

1,10-phenanthrolin-5-amine, 5-bromo-2-hydroxybenzaldehyde, and thiophene-3-carbaldehyde were procured from Sigma (Sigma, Mumbai, India), and S.D. Chemicals (RD Chem, Mumbai, India) and used without further purification. All the solvents were obtained from Merck (Goa, India), which were dried before using them in the experiments. Thin Layer Chromatography (TLC) was used to check the completion of reaction by spotting the reaction mixture on pre-coated silica-gel plates (Merck, Mumbai, India) and the spots were visualized by UV irradiation. Infrared spectral studies were performed on Perkin-Elmer spectrometer (PerkinElmer, Waltam, MA, USA) version 10.03.09 (KBr pellet technique). ^1^H and ^13^C NMR were recorded on a 500 MHz Bruker Avance DPX spectrometer (Bruker, Ettlingen, Germany) with TMS as internal standard. Mass spectra (ESI-MS) of the compounds were measured on Agilent mass spectrometer (Agilent, Santa Clara, CA, USA). The UV-Visible absorption spectra were recorded using UV-1800 spectrophotometer (Shimadzu, Koyto, Japan).

### 3.1. Biology

#### 3.1.1. Determination of Anticancer Activity of Synthesized Compounds

The in vitro anticancer activity of PIB and PTM was determined using the MTT assay. Breast cancer (MCF-7), Cervical cancer (HeLa), Colorectal carcinoma (HCT-116), Prostate cancer (PC-3) and Normal Human Fibroblast (HaCat) cells were procured from ATCC (Texas, USA). All the cells were cultured in DMEM added with 10% Fetal Bovine Serum (FBS), penicillin (100 IU/mL), streptomycin (100 µg/mL) in 5% CO_2_, at 37 °C, until confluent and cells were trypsinized, using 0.05% trypsin-EDTA solution to be checked for viability using a haemocytometer (Merck, Leicestershire, United Kingdoms). 100 µL of the media-diluted cell suspensions, containing 10,000 cells/well plated and incubated in 5% CO_2_ at 37 °C, till confluence. The cells were treated with 2.5, 5, 10, 20, 40 and 60 µM concentrations of PIB and PTM.

#### 3.1.2. Measurement of Cell Viability Using MTT Assay

MTT assay was performed according to DenizotandLang [21] published procedure. After 24 h, treatment cells were fixed using MTT reagent (5 mg/mL) to each well and incubated at 37 °C for 1 h followed by centrifugation at 3000 rpm for 5 min. Plates were removed from centrifuge and excess dye was washed and 100 μL of dimethyl sulphoxide (DMSO) was added to solubilize the crystal. Absorbance was read at 570 nm. Percentage of inhibition was calculated using the formula mentioned below and represented graphically using Prism 8 statistical analysis tool (GraphPad Software, San Diego, CA, USA).

% inhibition = [(OD of control − OD of sample)/OD of control] × 100(3)

### 3.2. Chemical Synthesis

#### 3.2.1. Synthesis of (*E*)-3-(((1,10-phenanthrolin-5-yl)imino)methyl)-5-bromophenol (PIB)

To a solution of 5-bromo-2-hydroxybenzaldehyde (75 mg, 0.37 mM) in 20 mL of dry methanol, about 100 μL of acetic acid was added drop wise to act as a catalyst during the reaction. To this above solution, 20 mg (0.10 mM) of 1,10-phenanthrolin-5-amine was combined with the help of a glass syringe. During this addition, color change was observed from colorless to pale brown solution. The above reaction mixture was then refluxed under continuous stirring at 60–65 °C for 4 h. After the completion of the reaction, the formed colored precipitate was filtered and washed several times with methanol to get desired product.

Yield: 186 mg (75.3%). m.p.: 234 °C. ^1^H NMR (CDCl_3_, 400 MHz, ppm): δ 8.94 (s, 1H), 8.03 (dd, 2H, *J* = 14 Hz), 7.46 (dd, 2H, *J* = 8 Hz), 7.25-6.58 (m, 7H, Ar-H). ^13^C NMR (100 MHz): δ 167.75, 139.36, 135.29, 133.46, 133.05, 132.81, 130.37, 128.88, 128.11, 128.08, 127.73, 127.68, 126.20 and 126.01. ESI-MS calculated for C_19_H_12_N_3_BrO [M]^+^: 377.02 and found is 377.10.

#### 3.2.2. Synthesis of (*E*)-*N*-(1,10-phenanthrolin-5-yl)-1-(thiophen-3-yl)methanimine (PTM)

To a solution of thiophene-3-carbaldehyde (83 mg, 0.42 mM) in 20 mL of dry methanol, about 100 μL of acetic acid was added drop wise to act as a catalyst during the reaction. To this above solution, 20 mg (0.10 mM) of 1,10-phenanthrolin-5-amine was combined with the help of a glass syringe. During this addition, color change was observed from colorless to yellow colored solution. The above reaction mixture was then refluxed under continuous stirring at 60–65 °C for 4 h. After the completion of the reaction, the formed colored precipitate was filtered and washed several times with methanol to get desired product.

Yield: 177 mg (72.7%). m.p.: 243 °C. ^1^H NMR (CDCl_3_, 400 MHz, ppm): δ 8.99 (s, 1H), 7.84 (dd, 2H, *J* = 4 Hz), 7.71 (dd, 2H, *J* = 6 Hz), 7.69–6.08 (m, 6H, Ar-H). ^13^C NMR (100 MHz): δ 167.51, 137.73, 136.91, 133.82, 132.35, 131.41, 130.09, 129.76, 129.00, 126.71 and 126.28. ESI-MS calculated for C_17_H_11_N_3_S [M]^+^: 289.07 and found is 289.80.

### 3.3. Statistical Analysis

All the experiments were conducted in triplicates and the results were expressed as mean ± SEM. The data were compiled into GraphPad Prism 8.0 software and were subjected to non-parametric unpaired t-test to determine the difference between the PIB and PTM treated groups and control. *p*-value of <0.05 was considered to be significant.

## 4. Conclusions

In summary, we have prepared two imine-based ligands, PIB and PTM, elucidated their molecular structures using spectroscopic methods. The prepared ligands were selectively cytotoxic against the breast (MCF-7), cervical (HeLa), colorectal (HCT-116) and prostate (PC-3) cancer cell lines, with no remarkable growth inhibition of the normal human keratinocytes (HaCat). Additionally, the cytotoxicity was dose and cell line dependent, with the highest concentration (60 μM) of PIB and PTM ligands inhibiting breast and colorectal, and cervical and breast cancer cell lines, respectively. In addition, using combination of DFT calculations and MD simulations, in this work we were able to identify specific reactive properties of PTM and PIB ligands. From the perspective of interactions with other molecules on the basis of electrostatics, MEP results indicate that the PIB ligand is more reactive than PTM, due to much higher maximal MEP values. While minimal ALIE values are practically the same for both ligands, the maximal ALIE value is higher in case of the PIB, indicating that higher amounts of energy are required for the removal of electron, finally meaning that PTM is slightly more reactive from the perspective of sensitivity toward the electrophilic attacks. The minimal H-BDE value is much lower in case of the PIB ligand. In fact, in case of the PIB, the minimal H-BDE value is also lower than the upper border threshold considered for the sensitivity toward the autoxidation mechanism. The PIB ligand is more reactive than the PTM ligand with respect to one more parameter, interactivity with water molecules. According to the MD simulations and calculated RDFs, the PIB ligand possesses atoms that have more significant interactions with water molecules. The OH group of the PIB is the most important in these terms, since hydrogen atom H35 has the maximal g(r) value located at the lowest distance, while the maximal g(r) value corresponding to the O35 atom is located at distance lower than 3 Å.

## Supplementary Materials

The following are available online at https://www.mdpi.com/1420-3049/25/12/2865/s1: ^1^H, ^13^C-NMR and mass spectra of the synthesized compounds.
