# Supplementary material for: Tumoricidal Potential of Novel Amino-1,10-phenanthroline Derived Imine Ligands: Chemical Preparation, Structure, and Biological Investigations
Source: Molecules. 2020 Jun 22;25(12):2865. doi: 10.3390/molecules25122865 (PMC7356530; doi:10.3390/molecules25122865)
Supplement: Supplementary file 1 [file molecules-25-02865-s001.pdf]

Supporting information

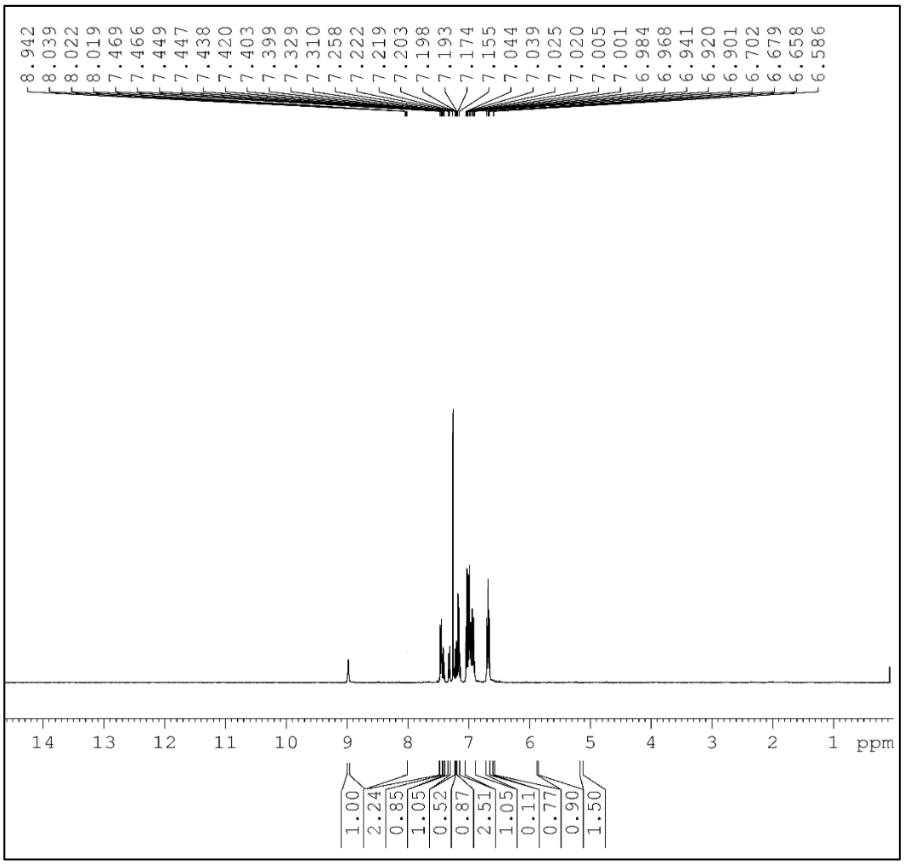

Figure S1: ^{1}H-NMR spectrum of PIB ligand.

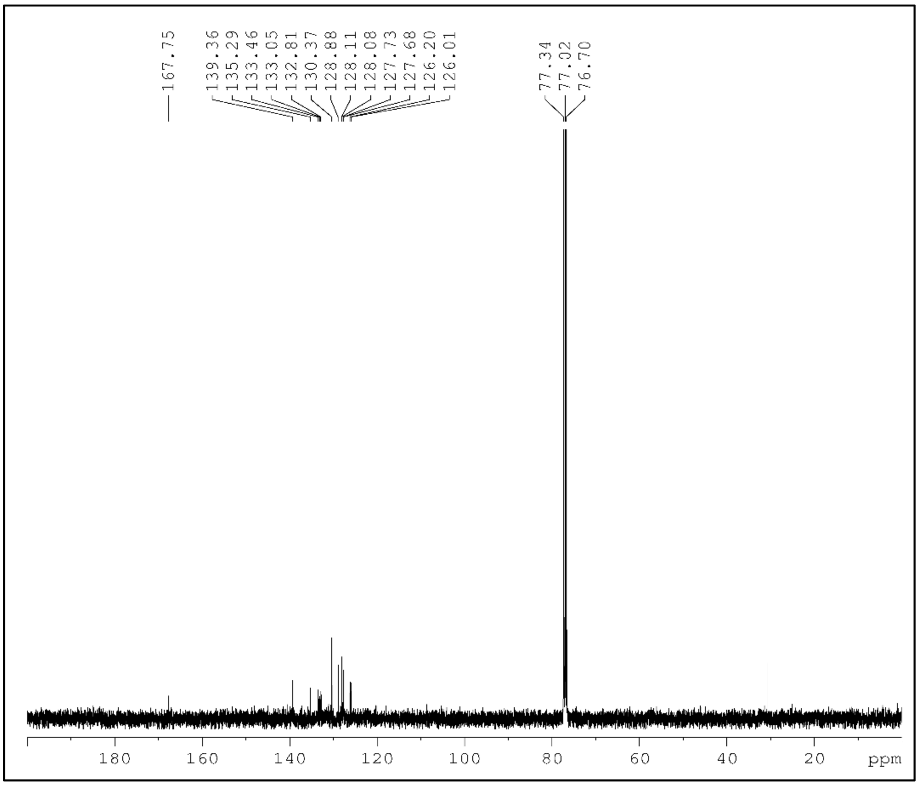

Figure S2: ^{13}C-NMR spectrum of PIB ligand.

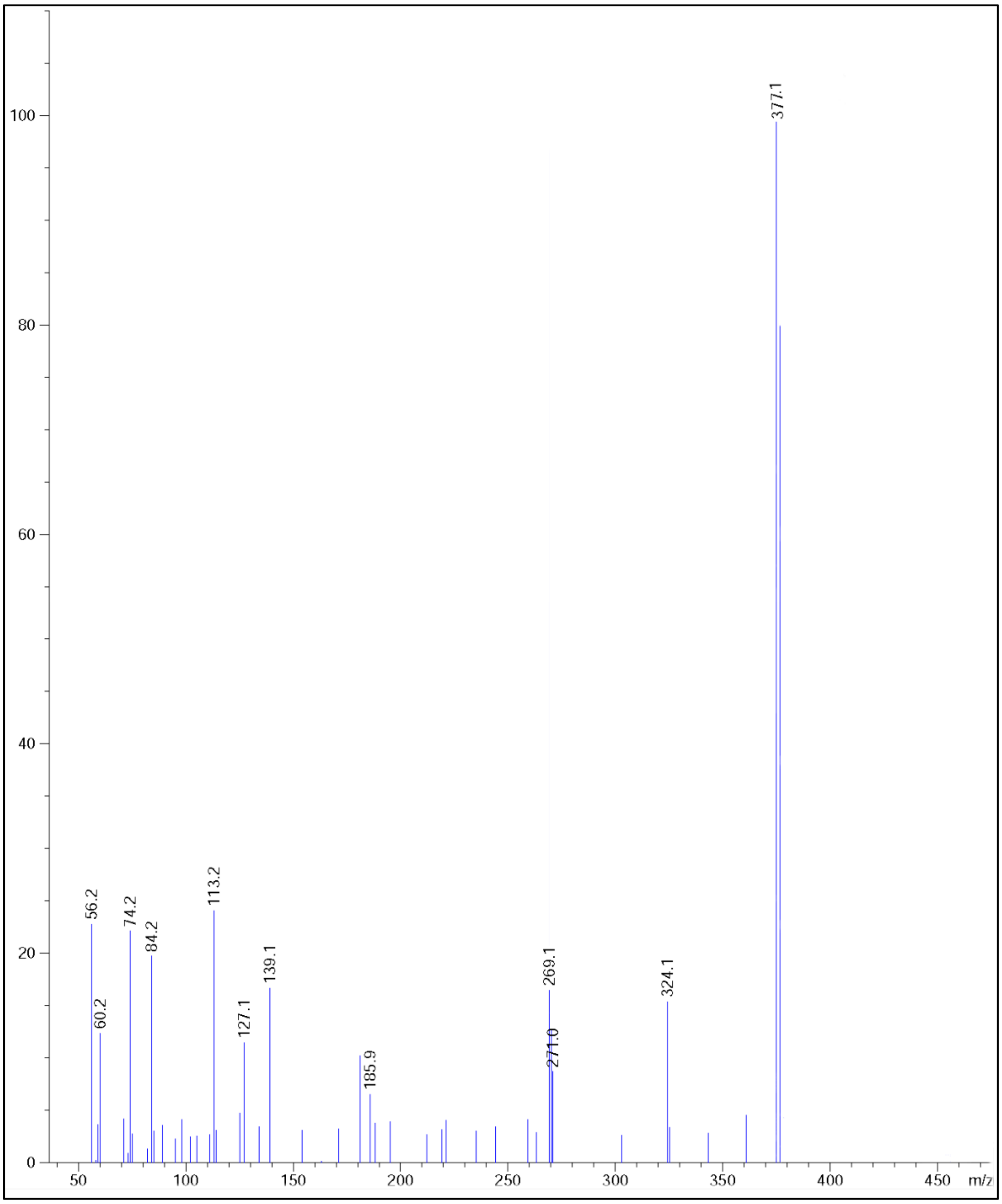

Figure S3: Mass spectrum of PIB ligand.

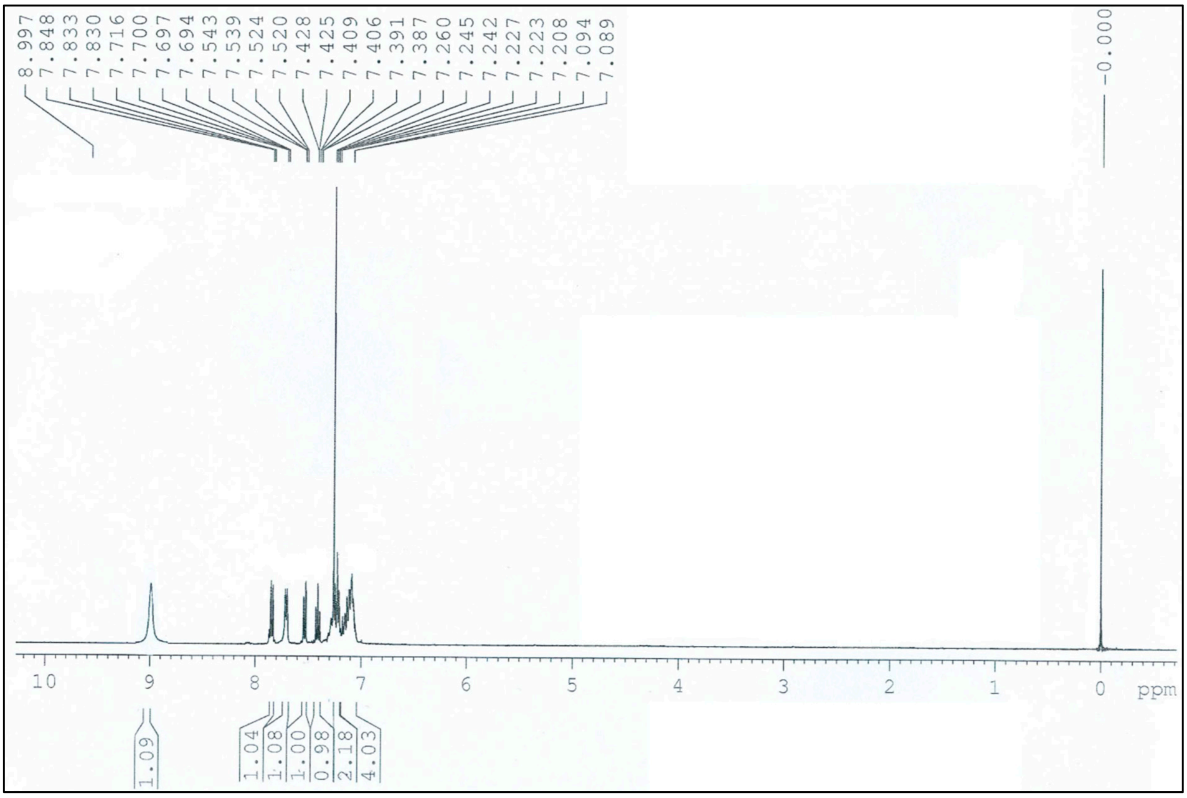

Figure S4: ^{1}H-NMR spectrum of PIB ligand.

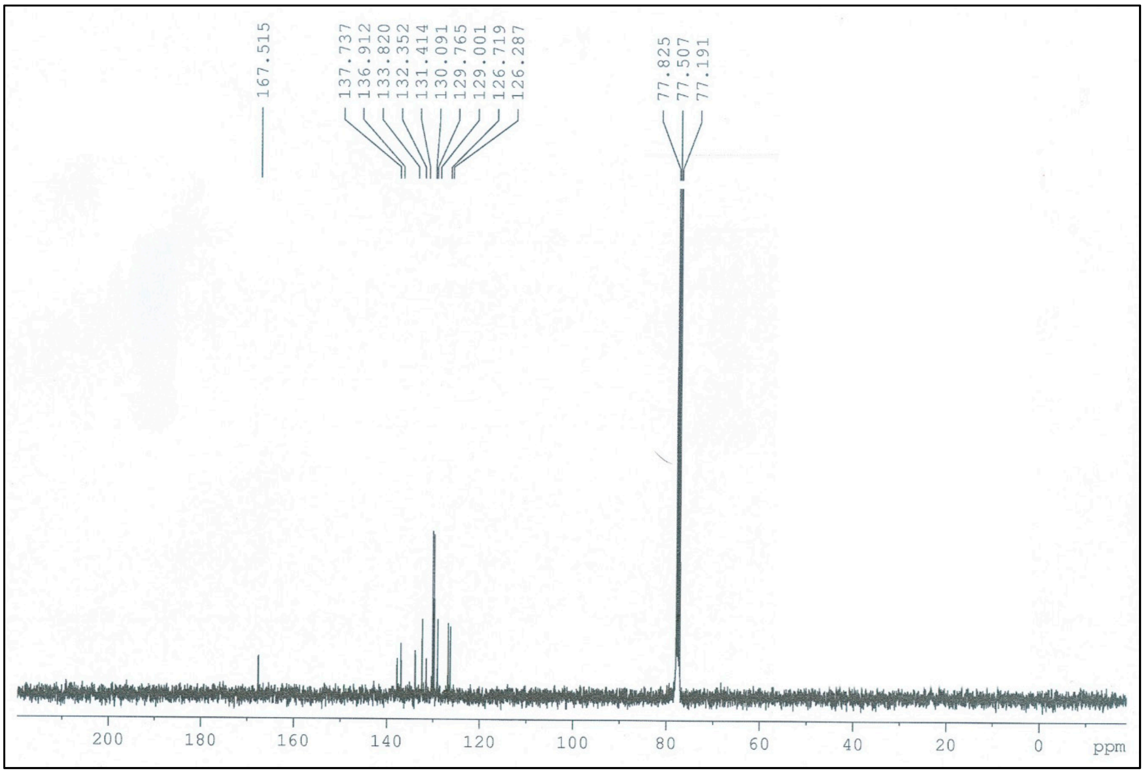

Figure S5: ^{13}C-NMR spectrum of PTM ligand.

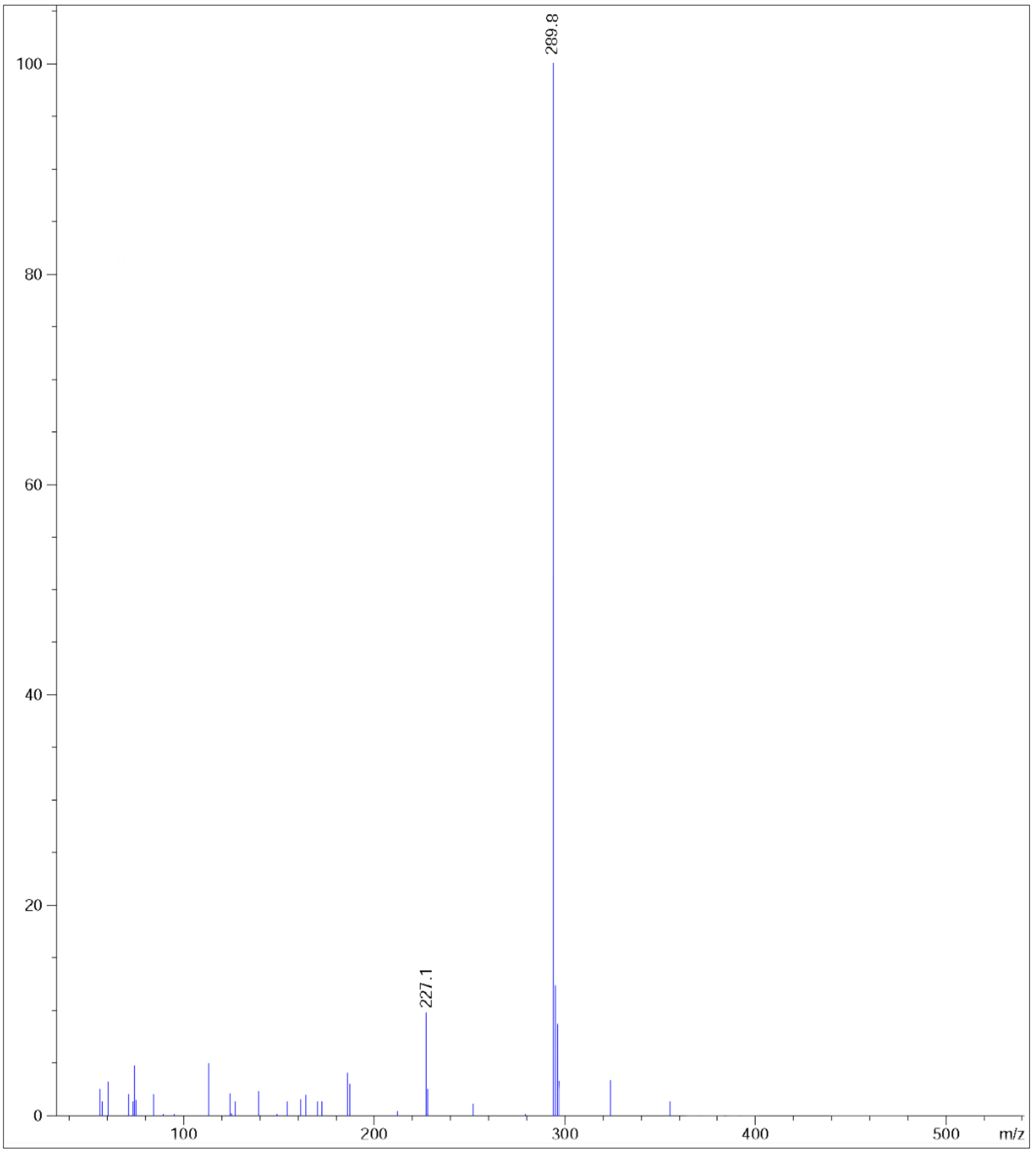

Figure S6: Mass spectrum of PTM ligand.
